# The role of Lagrangian drift in the generation of surface waves by wind

**DOI:** 10.1017/jfm.2026.11348

**Published:** 2026-03-30

**Authors:** L.R. Seitz, Mara A. Freilich, Nick Pizzo

**Affiliations:** 1 Division of Applied Mathematics, Brown University, Providence, RI 02912, USA; 2 Department of Earth, Environmental and Planetary Sciences, Brown University, Providence, RI 02912, USA; 3 Graduate School of Oceanography, University of Rhode Island, Narragansett, RI 02882, USA

**Keywords:** wind–wave interactions, surface gravity waves, air/sea interactions

## Abstract

A nonlinear stability analysis entirely in the Lagrangian frame is conducted, revealing the fundamental role of the wave-induced mean flow in modifying further wave growth and providing new insight into the classic problem of wave generation by wind. The prevailing theory, a critical-layer resonance mechanism proposed by Miles (*J. Fluid Mech*., 1957, vol. 3, no. 2, pp. 185–204), has seen numerous refinements; yet, the role of Lagrangian drift – the velocity a fluid parcel actually experiences – in wave growth was not understood. Our analysis first recovers the classic Miles growth rate from linear theory before extending it to third order in the wave slope to derive a modified growth rate. The leading-order wave-induced mean flow alters the higher-order instability, manifesting as a suppression of growth with increasing wave steepness for the realistic wind profiles considered. This modified growth rate shows good agreement with experimental observations, explaining the observed steepness-dependent suppression via a single physical mechanism. An integral momentum budget clarifies this mechanism, revealing that the wave-induced current alters the coupling between the total phase speed and the total Lagrangian mean flow at the critical level (as defined in the linear theory), thereby acting to reduce the efficiency of momentum transfer. Notably, this Lagrangian drift is precisely what Doppler-shift-based remote sensing of upper ocean currents measure, providing a direct observational pathway to account for this wave-induced feedback in studies of air–sea coupling. More broadly, this approach can be generalised to analyse other shear instabilities and provides a direct path towards refining wind-stress parametrisations.

## Graphical abstract

**Figure:**
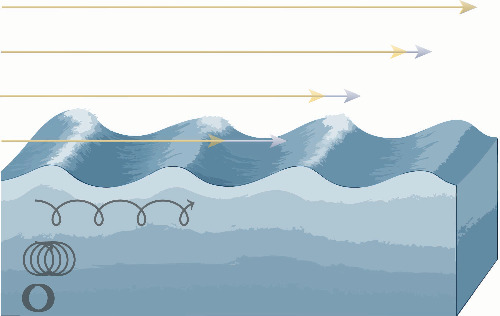

## 1. Introduction

The generation of ocean waves by wind remains a foundational problem in fluid dynamics, though it has evolved considerably since Miles’ (1957) pioneering work establishing that instability due to wind shear constitutes a fundamental physical mechanism for wave growth. Subsequent efforts have developed in complementary yet divergent directions: enhancing the understanding of air–sea momentum transfer by detailing the influence of specific factors – such as shear flow in the water (Valenzuela 1976; Young & Wolfe 2014), viscous stresses (Benjamin 1959; Miles 1959) and airflow dynamics (Riley, Donelan & Hui 1982; Al-Zanaidi & Hui 1984; Grare *et al.* 2013*a*; Buckley, Veron & Yousefi 2020; Cao & Shen 2021) – beyond Miles’ (1957) original framework, while simultaneously pursuing simplified representations of wind stress to incorporate into ocean models (Plant 1982; Weber 2003; Drennan *et al.* 2003). Yet, this body of work has struggled to resolve key discrepancies found in experimental data (Sullivan & McWilliams 2010).

It is well established that waves influence momentum transfer into the upper ocean, through both wave-induced Reynolds stresses (Van Duin & Janssen 1992; Miles 1993) and the wind-induced current prior to wave generation (Young & Wolfe 2014). Beyond these interfacial effects, the interaction between surface waves and shear flow can generate Langmuir circulation, which drives an efficient downward redistribution of horizontal momentum and tracers from the surface (Thorpe 2004; Belcher *et al.* 2012; Hamlington *et al.* 2014; Li *et al.* 2017; Wagner *et al.* 2023). However, the full role of the wave-induced current in the instability mechanism itself has remained poorly understood. Addressing this theoretical gap yields a new explanation for the systematic dependence of wave growth rates on mean wave steepness found in laboratory experiments and indicated by observations (Peirson & Garcia 2008). These considerations lead to the central question of the present work: does the wave-induced mean current play a role in the resonance interaction underlying the physical mechanism behind wave growth? Answering this question also holds the potential to provide a new perspective where the influence of other factors, e.g. surface roughness or induced Reynolds stresses, on wave growth is understood through their intrinsic connection to the wave-induced mean flow.

In the classic theory, Miles established that the growth of surface gravity waves due to wind can be predicted based on a resonance interaction between the intrinsic frequency 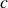 of an initial sinusoidal disturbance on the water surface and the leading-order Eulerian mean velocity 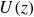 at a critical level 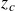 in the air. Yet, the wavy disturbance creates a distinction between Eulerian and Lagrangian descriptions of the mean flow, perturbing the mean motion of parcels in the bulk of both the air and the water. This difference between the Lagrangian-averaged velocity and the Eulerian-averaged velocity is the Stokes drift (Andrews & McIntyre 1978), 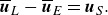 Miles (1957) used 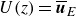 at leading order. The question of the role of the wave-induced current in the instability can thus reduce to understanding the role of the full Lagrangian mean. This is further motivated by recent findings showing that (i) remote sensing techniques based on observing current-induced shifts in the wave dispersion measure the Lagrangian, rather than Eulerian, mean current (Pizzo *et al.* 2023); (ii) Stokes drift likely contributes significantly to upper ocean shear (Lenain *et al.* 2023); and (iii) including a Stokes drift term when modelling the time evolution of nonlinear surface waves avoids significant errors in the wave celerity (Guérin *et al.* 2019).

Recent advances in Lagrangian theory highlight a promising approach for addressing these questions. These advances include a rigorous asymptotic expansion for the Lagrangian description of steady surface gravity waves (Clamond 2007), the development of a second-order Lagrangian description of surface gravity wave interactions that captures nonlinear phenomena absent in Eulerian descriptions of the same order (Nouguier, Chapron & Guérin 2015), clarification of how Lagrangian drift influences the geometry, phase speed and stability properties of surface waves (Pizzo *et al.* 2023), and the demonstration that Lagrangian drift may be identified with mean Lagrangian momentum density and is determined by vorticity (Blaser *et al.* 2024). Despite these advances, the role of Lagrangian drift in the instability is *a priori* unclear. Although the Rayleigh equation has been formulated in the Lagrangian frame (Bennett 2006), which was recently used to analyse barotropic and baroclinic instabilities (Bennett 2025), we present the first higher-order analysis of any hydrodynamic shear instability entirely in the Lagrangian frame. This methodology also addresses the absence of formal Lagrangian analysis in the physical understanding of the instability evolution, which is essentially a Lagrangian parcel argument (Lighthill 1962).

While the Eulerian and Lagrangian reference frames are theoretically equivalent, and thus it should be possible to recover the effects discussed in this work in an Eulerian framework, the Lagrangian framework offers distinct advantages for analysing wave-induced currents. Prior studies used Lagrangian equations of motion to formulate evolution equations for wave-induced mass transport in the ocean, leading to alternative models for wave growth, albeit in a context different from the Miles mechanism (Weber 1983; Jenkins 1986; Weber & Melsom 1993). A Lagrangian approach thus holds the potential to resolve both the theoretical question of how Lagrangian drift influences wave generation and the practical question of which reference frame is best for wind stress estimation. Moreover, this perspective can reveal physical interpretations that are hidden in an Eulerian view.

In § 2, by asymptotically expanding Lagrangian trajectories in terms of permanent progressive monochromatic waves, we demonstrate that it is possible to conduct the stability analysis as done by Miles (1957) and Young & Wolfe (2014) in the Lagrangian frame. While our analysis recovers the classic growth rate (of the linear theory), additional insights about its spatial dependence and the shape of the critical layer emerge due to the Lagrangian perspective. In 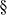 3, we extend the analysis through third order in the wave slope to derive a modified instability growth rate, showing how it is altered by the (leading-order) wave-induced mean flow. In addition, we derive the formula for the curvature of the free surface in Lagrangian coordinates and the corresponding higher-order correction to the growth rate due to surface tension, extending the classical theory to account for nonlinear wave–mean flow interactions in the presence of capillarity. In § 3.3, we approximate the growth rate in the case of the more realistic background logarithmic profile. This allows us to compare the results with experimental data in § 3.4, showing that the modified growth rates reproduce the trend of steepness-dependent suppression found in observations. We interpret the physical mechanism behind the suppression by formulating an integral momentum budget in § 3.5. The momentum budget demonstrates that the interaction between the total phase speed 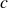 (summing contributions at all orders of the wave slope) and the total Lagrangian mean flow 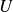 plays a critical role in the net momentum input from the wind. This confirms that the instability is dependent on the complete, wave-altered velocity field that fluid parcels experience, consistent with qualitative arguments from prior work. Furthermore, these inferences support the view that the growth modification that appears once the wave-induced mean flow is accounted for acts as an intrinsic mechanism for the previously observed suppression. Lastly, in § 4, we discuss the broader implications of our Lagrangian analysis, including the applicability of this framework to other shear instabilities, generalisations to this framework and the consequences for estimating air–sea momentum transfer.

## 2. Formulation of the linear stability problem in Lagrangian coordinates

### 2.1. Lagrangian governing equations

In the Lagrangian frame, fluid particles (or parcels) are tracked using fixed labels 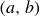 and time 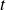 as independent variables. Time derivatives are taken with respect to fixed particle labels 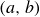 . Trajectories are then described by 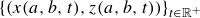 . The mapping between label space 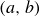 and physical space 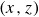 is invertible, as no two particles can occupy the same physical location simultaneously. This implies a non-zero Jacobian:
(2.1)

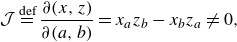

where 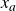 denotes the partial derivative of the 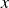 -coordinate with respect to particle label 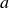 , and similarly for the other derivatives. While this mapping is generally time-dependent, our choice of labels based on particle locations at time 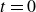 ensures that for an incompressible fluid, the Jacobian is constant along a trajectory, i.e. 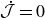 . Beyond these constraints, there remains considerable flexibility in particle label assignment.

We choose particle labels such that the free surface corresponds to 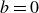 . We assign labels based on particle positions at time 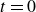 , with label 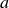 following the 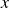 -axis. Our two-fluid system consists of water ( 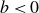 ) and air 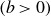 . This Lagrangian formulation offers a significant geometric advantage: the time-varying free surface can be described as a simple, fixed plane in label space rather than an additional function 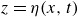 . The conservation of particle labels implies that particles at the surface remain at the surface for all time. This approach directly circumvents the well-known difficulties of defining physically meaningful averages in the Eulerian frame near an undulating interface, a challenge that has motivated the development of analogous coordinate transformations in experimental and numerical work (e.g. Sullivan, McWilliams & Patton 2014; Buckley & Veron 2016). Furthermore, this formulation has a significant analytical advantage in that Lagrangian approximations are generally more accurate and converge faster than Eulerian ones of the same order. For moderately steep waves, for instance, an 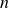 th-order Lagrangian approximation of the surface can match the accuracy of an Eulerian approximation of order 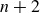 (Clamond 2007).

The Euler equations in Lagrangian coordinates are given by (Lamb 1924, Art.15) 
(2.2a)

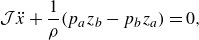



(2.2b)

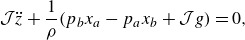




where 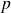 is pressure, 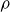 is density and 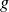 is the gravitational constant. Since particle density remains constant along trajectories, we take 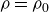 as constant.

Lastly, the vorticity is defined as
(2.3)

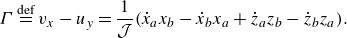

Vorticity is always conserved, so that 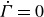 . For irrotational flows, 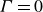 .

As in the classical stability analysis (Miles 1957), we neglect viscosity and turbulent stresses, and model the evolving perturbation as a normal mode on a prescribed, smooth background shear 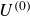 . We thus interpret the theory as most applicable to non-breaking conditions in which the airflow remains predominantly attached and the wave steepness is small, 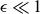 . Laboratory observations indicate that airflow separation is uncommon over low-wind, small-slope waves, but becomes increasingly prevalent as wind forcing and wave slope increase (Buckley *et al.* 2020). Within this attached-flow regime, we isolate the wave-induced mean flow as the leading higher-order correction, yielding the order 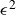 modification to the Miles growth rate derived in 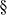 3.

### 2.2. Asymptotic expansions

We consider permanent, progressive, monochromatic, spatially periodic, two-dimensional waves progressing with velocity 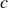 (Clamond 2007; Pizzo *et al.* 2023). The coordinates 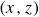 are expanded as
(2.4)

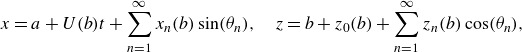

where 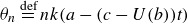 , 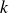 is the wavenumber and 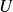 is the Lagrangian mean flow (and may be identified with 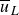 evaluated in Lagrangian coordinates). The mean surface level is 
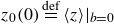
 , where 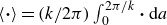 denotes phase averaging. Pressure is expanded as
(2.5)

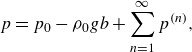

where 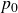 denotes the constant background pressure, so that 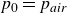 in the air and 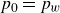 in the water. As done by Pizzo *et al.* (2023), the pressure can be solved as
(2.6)

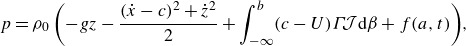

where 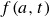 is a constant of integration.

Imposing ordering in (2.4), through third order in the wave slope 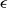 , 
(2.7a)

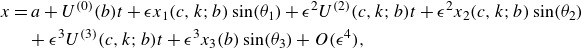



(2.7b)

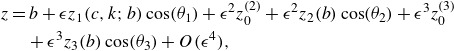



(2.7c)

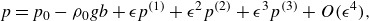




where the total Lagrangian mean flow has been written as 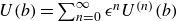 , so 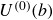 denotes the leading-order portion and 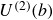 is associated with the Stokes drift. The terms 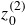 and 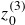 in 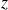 are to ensure 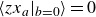 (note that 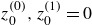 ). Additionally, the phase speed is expanded as 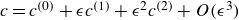 . The phase speed is also expanded in this manner by Clamond (2007), and this expansion makes it possible to find the modified growth rate in § 3.

### 2.3. Instability growth rate and critical level in Lagrangian coordinates

The linear stability analysis, conducted in the Lagrangian frame, recovers the established Miles instability growth rate. This approach, however, offers a more intuitive understanding of the critical level’s geometry, showing it is a wavy surface that follows the interface, which is less apparent in a traditional Eulerian analysis. To calculate the instability growth rate, we first formulate the Rayleigh equation in Lagrangian coordinates, which requires a change of variables: 
(2.8a)

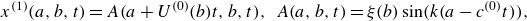



(2.8b)

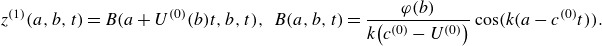




The Rayleigh equation depends only on 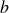 and is derived from the linear approximations of (2.2a)–(2.2b) and mass continuity (see Appendix A) as
(2.9)

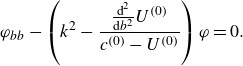




We will use the Rayleigh equation after deriving a dispersion relation from the linearised dynamic boundary condition. In Lagrangian coordinates, the linearised dynamic boundary condition at the air–sea interface is given by
(2.10)

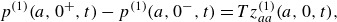

where 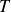 is the coefficient of surface tension.

Denote 
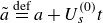
 , where 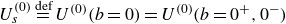 . In the new variables (2.8*b*), using (2.6), (2.10) reduces to
(2.11)

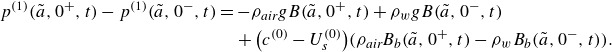

Define the constants
(2.12)

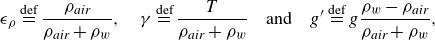

and the functions 
(2.13a)

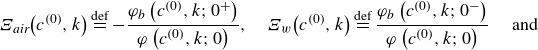



(2.13b)

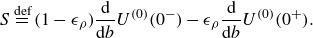




After substituting (2.10) into (2.11) and some algebra, we obtain the dispersion relation
(2.14)

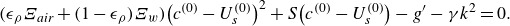

As done by Young & Wolfe (2014), we rewrite (2.14) as
(2.15)

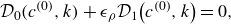

where
(2.16)

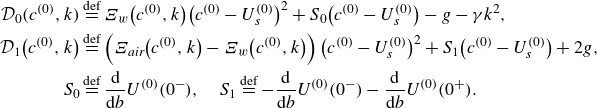

The solution to (2.15) will be of the form 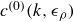 , so we expand
(2.17)

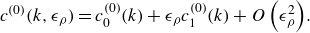

We then obtain the leading-order balance
(2.18)

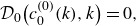

which only involves flow in the water, so does not itself affect the Miles instability. The Miles instability results from a critical level in the air, 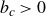 , such that 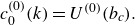 At next order in 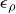 , using a Taylor expansion in terms of 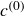 , we can solve
(2.19)

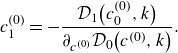




The instability growth rate is that of the unstable solutions to the eigenvalue problem. Notice the Rayleigh equation (2.9) becomes singular when 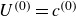 ; to avoid this singularity, we introduce a complex phase speed
(2.20)

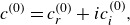

where 
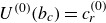
 and 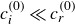 . The form of the perturbation in (2.8*b*) grows in time according to 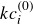 , so that the instability growth rate (in this linear analysis) is 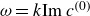 . Noting that 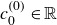 , we approximate the growth rate 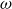 as
(2.21)

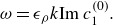

We then compute that
(2.22)

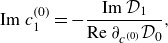

where (see Appendix A)
(2.23)

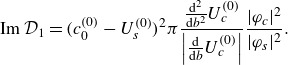

Here, 
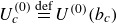
 , 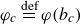 and 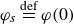 .

By inverting 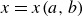 and 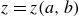 iteratively at each order of 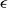 , using (2.7*a*) and (2.7*b*), we can write 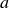 and 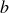 in terms of 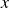 and 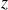 : 
(2.24a)

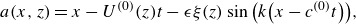



(2.24b)

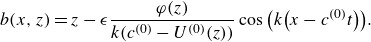




We thus recover the known growth rate for the Miles instability, but in the Lagrangian frame; 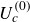 and 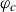 are functions of 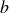 rather than 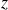 , but to leading order 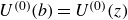 and 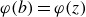 , per (2.24*a*) and (2.24*b*). However, the representation of the growth rate (2.21) given by (2.22) and (2.23) differs from the Eulerian representation because the arguments of 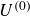 and 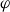 should be evaluated at the perturbed 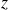 -coordinate described by the right-hand side of (2.24*b*), rather than simply 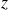 . The critical level, defined by a constant 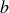 (or equivalently, perturbed 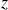 ), is therefore ‘wavy’, following the shape of the interface. Indeed, (2.24*b*) shows that a line of constant 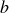 is not one of constant physical height 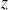 , but rather a wavy surface oscillating in 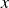 (figure 2). Note that at the linear order considered here, the waviness of the critical level is a geometric consequence of the mapping (2.24*b*) and does not introduce an additional phase offset beyond the classical Miles mechanism, as reflected in the recovery of the standard linear growth rate. The linear growth rate varies across the interface, since 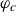 and 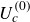 in (2.23) occur at a constant level 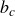 , which is not a constant height 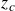 per (2.24*b*). The waviness of the critical layer, stated by Lighthill (1962) and apparent through the use of orthogonal coordinates by Benjamin (1959), emphasises that the critical height 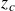 is better regarded as height from the actual, rather than mean, water surface.

This explicit description of the geometry of the critical level is one advantage of the Lagrangian analysis. A more significant outcome of this analysis, however, is the Lagrangian frame’s capacity to show how the wave-induced mean flow modifies the instability growth rate, a task that requires extending the stability analysis to higher order.

## 3. Modification to instability growth rate by the wave-induced mean flow

### 3.1. Nonlinear stability analysis

Computing the modification to the growth rate by the wave-induced current requires considering the system through third order. This is similar to other nonlinear stability analyses, e.g. Nayfeh & Saric (1972), but in the Lagrangian frame, we expand in terms of monochromatic waves (2.4) and expand the phase speed and mean velocity (e.g. Clamond 2007) and not the independent variables 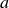 , 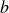 or 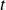 . In the higher-order analysis, we first consider the special case in which 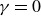 in (2.12), as in Miles’ original analysis. The full details of the higher-order analysis are provided in Appendix B. While it turns out that 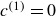 due to the second-order conservation of vorticity, 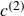 is described by the dispersion relation
(3.1)

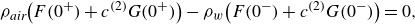

where 
(3.2a)

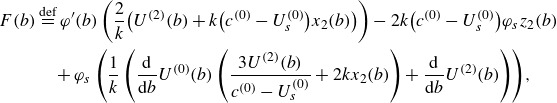



(3.2b)

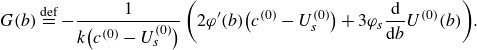




In contrast to our approach to obtain the linear growth rate, since the dispersion relation (3.1) is only linear in 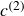 , we obtain a relatively simple analytical solution without using an asymptotic expansion in 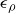 :
(3.3)

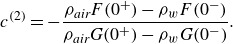

Additionally, under the mild assumption that derivatives of 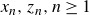 introduce a constant coefficient of 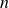 (as in a Gerstner or Stokes wave), an analytical formula for 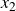 and 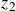 in terms of the known lower order quantities 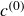 , 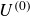 and 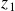 is possible,
(3.4)

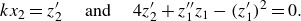

The leading-order wave-induced mean flow 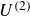 can also be expressed in terms of known lower-order quantities (see Appendix E),
(3.5)

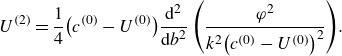

Thus, (3.3) is entirely in terms of known quantities, since we can also use the linear growth rate when considering 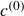 , (2.21). However, it may not be computable in general, as 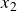 may be an indefinite integral that cannot be computed. Nonetheless, (3.3) readily gives an exact solution for the modified growth rate for many relevant background wind profiles 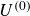 . Regardless of the ability to compute an exact solution, the dispersion relation clearly indicates that the leading-order growth-rate modification will be directly affected by the wave-induced mean flow, due to the appearance of 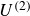 throughout, as well as its shear.

### 3.2. Growth rates with respect to the double exponential profile

To illustrate the impact of the modified growth rate, we consider an explicit base-state velocity profile
(3.6)

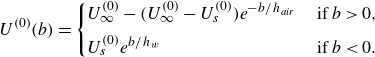

This double exponential profile is defined by the scale heights in the air and water ( 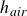 and 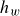 ), and the velocities at the top of the boundary layer and at the surface ( 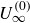 and 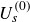 ). For a given profile, the Rayleigh equation has an exact solution
(3.7)

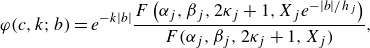

where the parameters to the Gaussian hypergeometric function 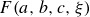 are defined as
(3.8)

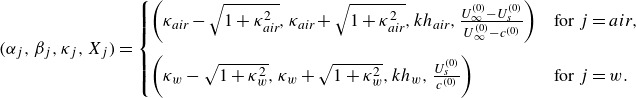

The amplitude corresponding to a given background shear profile therefore depends on both the wavenumber 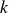 and the parameters in the definition of the background profile.

The Gaussian hypergeometric function in (3.7) is given by
(3.9)

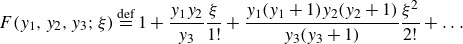




**Figure 1.**
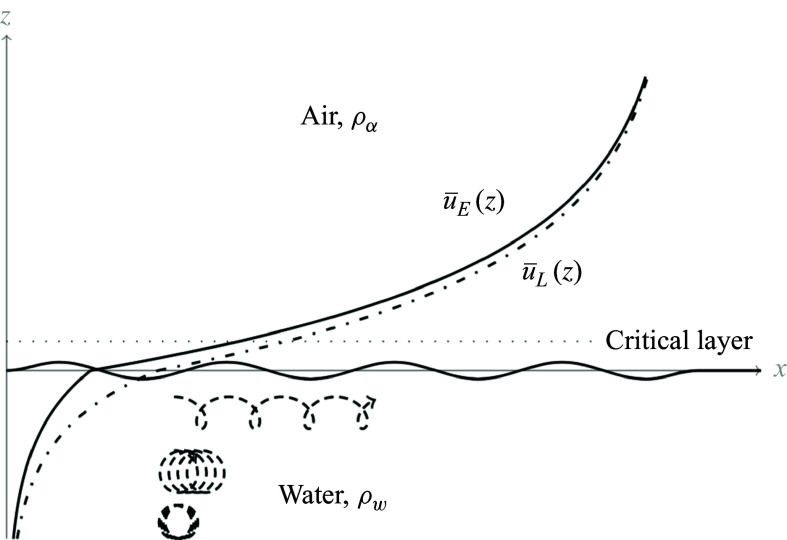
The set-up of the Miles instability in Lagrangian coordinates, adapted from Young & Wolfe (2014). The Lagrangian mean velocity profile, 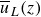 (identified as 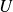 in (2.4)), is shown alongside the Eulerian mean, 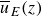 . While the two profiles are identical at leading order, they differ at higher order due to the surface wave; note that 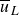 is more naturally a function of the particle label 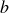 , but is shown in terms of 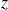 for comparison. Example trajectories (dashed), with the same initial 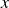 -coordinate, are in the water. As the Lagrangian mean velocity approaches zero, the orbits of particles become closed circles.

In the case of this double exponential profile (3.6) (figure 1), 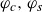 , 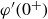 and 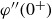 may be computed exactly using (3.7), the differentiation identity for hypergeometric functions, and the Rayleigh equation, respectively. In addition, if 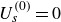 , as in the original case considered by Miles (1957), (3.3) may be explicitly, exactly computed, whereas more complex background profiles may necessitate approximation via numerical methods. Assuming 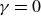 ((2.12), no surface tension) also as in the original work, the growth rate (2.21) is evaluated as
(3.10)

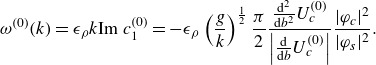




**Figure 2.**
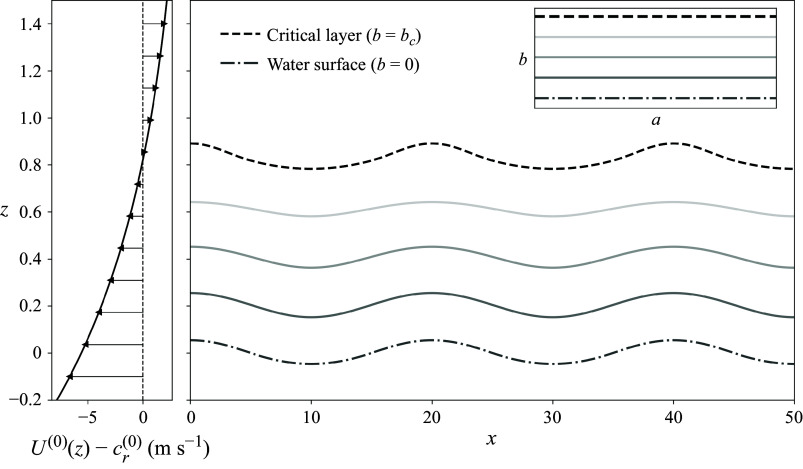
The critical layer defined by 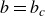 , the surface defined by 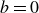 and intermediate lines of constant 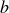 , shown in Eulerian coordinates (the 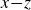 plane) by using the transformations (2.24*a*) and (2.24*b*). These levels of constant 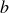 are also shown in the Lagrangian frame (the 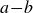 plane) in the inset, illustrating the simple representation of the wavy critical level when considering the instability in Lagrangian coordinates. The relative wind is shown on the left. Here, the background wind is the double exponential profile described in § 3.2 with 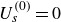 (the original case Miles considered, together with 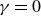 ), 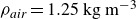 , 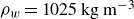 , 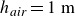 and 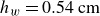 . In this case, 
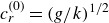
 , where a wavelength of 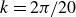 and a wave slope of 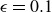 were selected (arbitrarily) for visualisation. This picture would evolve in time according to (2.24*b*); here, 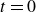 is shown.

**Figure 3.**
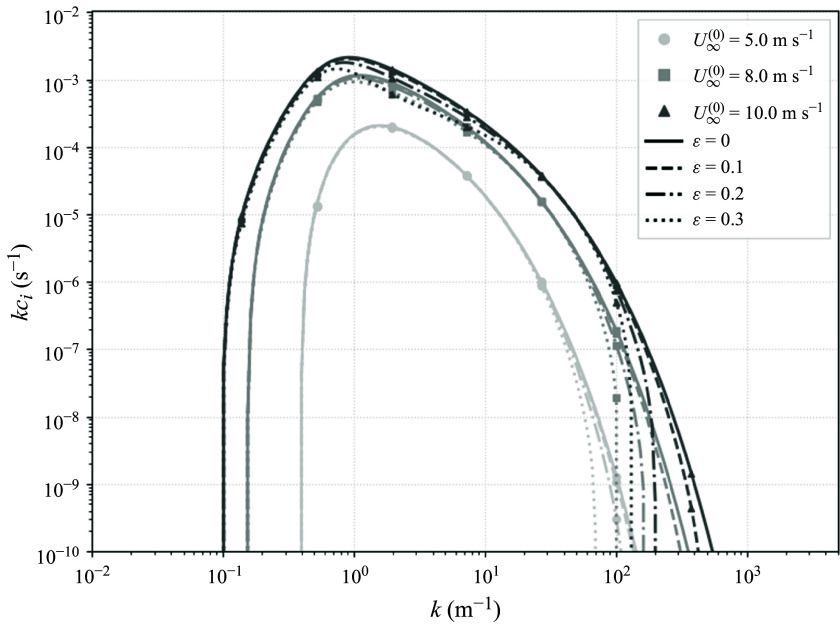
Modified growth rate as a function of wavenumber 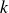 , for four wave slopes 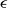 and three values of 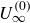 (which increases the background shear). The linear growth rate ( 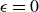 , solid) computed in the Lagrangian frame equals the known result computed in the Eulerian frame, while the modified growth rates, dependent on the leading-order wave-induced mean flow ( 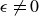 ), show that increased wave slope combined with increased background shear can lead to a significant suppression of the instability at high wavenumbers. For larger values of 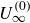 , the modification can also significantly reduce the peak growth rate. Growth rates were computed for the double-exponential profile with parameters: 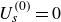 , 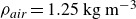 , 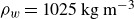 , 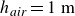 and 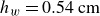 (as in Young & Wolfe 2014). The values of 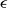 chosen are based on observational data, e.g. those of Peirson & Garcia (2008). The linear growth rate shown here is the asymptotic solution, but it exactly aligns with the numerical solution to (2.14) (cf. Young & Wolfe 2014).

In total, the modified growth rate is given by (3.10) and (3.3) via
(3.11)

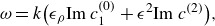

which is plotted for different values of 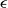 in figure 3. Note that the 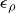 and the subscript 1 are due to the asymptotic expansion done to find 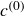 , which is unnecessary when computing 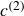 . The formula for 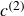 (3.3) also incorporates the relative densities. Consistent with linear theory (figure 3, solid curves), the growth rate peaks at an intermediate wavenumber and increases with background shear (modified via increasing 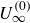 ). For a finite but non-zero wave slope 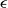 , this growth is systematically suppressed, an effect that intensifies with increasing 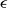 . For moderate values of 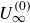 , this suppression is largely confined to high wavenumbers, but at larger 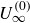 values, it extends even to intermediate wavenumbers, reducing the peak growth rate by as much as a factor of four for the largest wave slope. The aggregate modulation, obtained by trapezoidal integration of the growth curves shown in figure 3, exhibits a maximum growth rate decrease (among the parameters shown) of 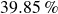 for the 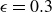 , 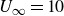 case and a minimum decrease of 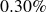 for the 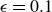 , 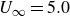 case.

**Figure 4.**
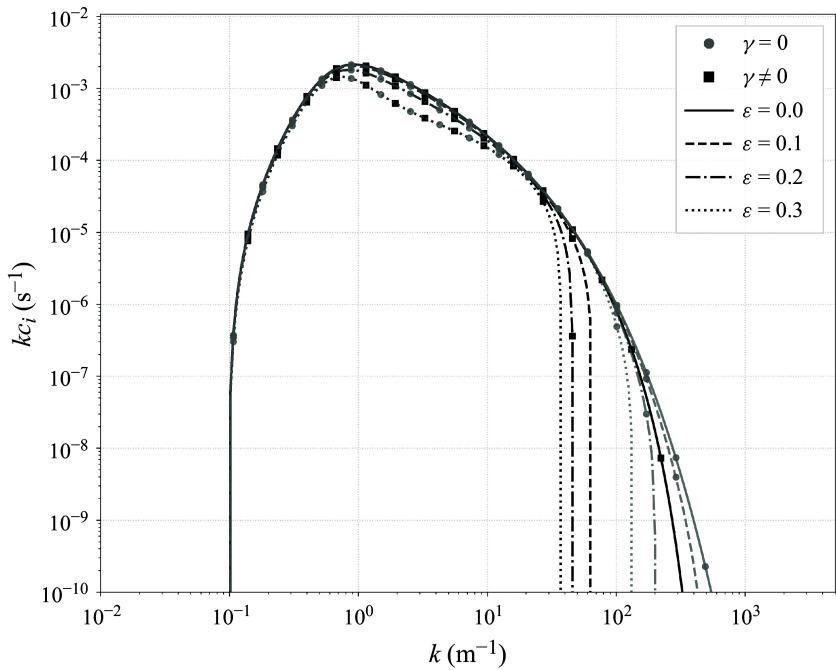
Modified growth rate with capillarity as a function of wavenumber 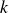 , for four wave slopes 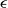 . The grey curves are as in figure 3 ( 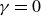 ) whereas the black curves show the impact of including surface tension effects ( 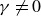 , here 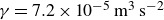 ). The parameters are as in figure 3, but all growth rate curves are with respect to 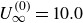 m s 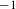 . In addition to the modification from the wave-induced current, surface tension further suppresses growth at high wavenumbers. However, surface tension does not alter the effect of the modification to growth rate shown in figure 3 at intermediate wavenumbers.

Up to this point, we have considered the same system as Miles (1957) and neglected the effects of surface tension. Since the predicted impacts of the higher-order theory are largest for shorter waves (figure 3), which are likely impacted by surface tension, we also compute the impacts of surface tension on both 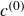 and 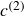 . The inclusion of surface tension does not change that 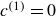 . The full calculation of these effects involves computing the curvature at points on the surface in Lagrangian coordinates by considering it as a parametric curve 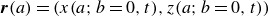 and expanding asymptotically, and is included in Appendix C. A typical value of the kinematic surface tension 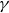 is 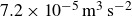 and the modified growth rate computed with this value is shown in figure 4. While the intermediate wavenumbers affected by the modified growth rate (i.e. the inclusion of the wave-induced current) have the same growth whether surface tension is included or not, the highest wavenumbers ( 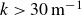 ) are significantly impacted by capillary effects. The impacts of surface tension, like those of the inclusion of the wave-induced mean flow, increase with wave slope. In all, the stabilising effect of surface tension for short waves is amplified by the nonlinear modification of the growth rate shown in figure 3.

### 3.3. Growth rates with respect to a logarithmic profile

While the double exponential profile conveniently permits analytical solutions, and thus more accurate solutions for the growth rate, it is representative of laminar flow; in contrast, a background logarithmic profile approximates a (mean) turbulent wind velocity, providing a realistic model better suited for comparing growth rates against empirical data (Morland & Saffman 1993). To that end, we extend Miles’ (1957) method for approximating the growth rate given a background logarithmic profile
(3.12)

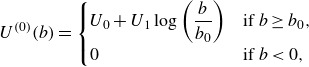

to our Lagrangian, higher-order analysis. In (3.12), 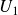 is a reference wind speed, 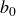 is a label near 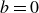 (analogous to the near-surface streamline in Miles’ analysis) and 
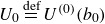
 (see Appendix F). We explicitly leave the profile undefined in the viscous/transition layer 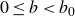 ; as done by Miles (1957), we assume this layer ‘moves with the surface wave’, and treat 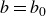 as the effective air-side interface. We then evaluate air-side quantities at 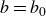 and water-side quantities at 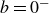 , in the growth rate approximation.

It is often preferable, especially when comparing against empirical data, to rewrite (3.12) in terms of an aerodynamic roughness length 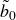 ,
(3.13)

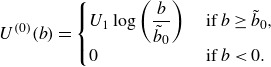

The representations (3.12) and (3.13) are equivalent provided 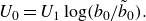 The near-surface label 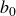 may then be chosen in relation to the aerodynamic roughness 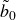 , e.g. 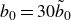 as in the classical case (Miles 1957, following (7.5*a*,*b*)).

To obtain the leading-order growth rate (in Eulerian coordinates), Miles (1957) assumes a particular form of the pressure that is proportional to a dimensionless coefficient, i.e. 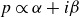 , where 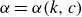 and 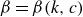 . For consistency with that presentation, we will then find the higher-order expression for 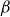 , which is a normalised growth rate proportional, but not equal, to 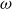 . For instance, 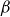 equals 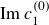 in (3.10) (Miles 1957, (3.3)). It is also assumed that the real part of the phase speed is not significantly altered by the process of interest, so that
(3.14)

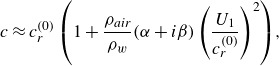

with 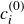 and 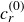 again as in (2.20). Miles then finds that
(3.15)

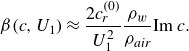

When extending (3.15) to account for not only 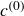 but also 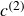 , to remain consistent with the original approximation, one must still assume that
(3.16)

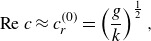

and thus simply substitute in 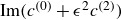 for 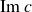 in (3.15). Substituting (3.12) into all relevant quantities in the definition of 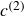 (3.3) yields
(3.17)

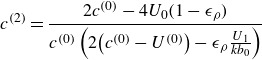

(see Appendix F). To find the imaginary part of (3.17), we expand 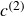 in terms of 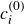 , akin to (2.17), which yields a simple form for 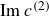 ,
(3.18)

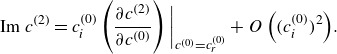

Evaluating (3.18) per (3.17) gives the approximate modified growth rate
(3.19)

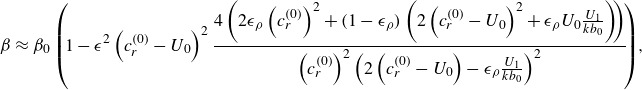

with 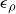 as in (2.12). In (3.19), 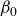 is the leading-order approximate growth rate, obtained by translating the calculation of 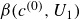 from Miles (1957) into Lagrangian coordinates,
(3.20)

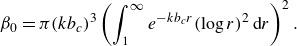

In (3.20), one may calculate from (3.12) that the critical level is 
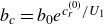
 .

**Figure 5.**
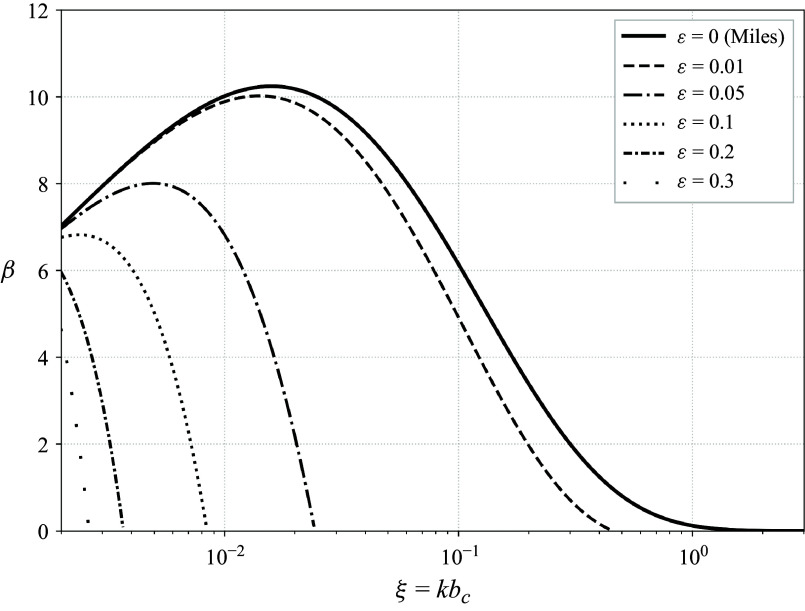
Normalised growth rate 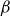 as a function of the dimensionless critical level 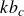 . All curves are calculated per (3.19). The solid line ( 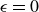 ) is an exact reproduction of figure 1 of Miles (1957). The dashed, dash-dotted and dotted curves show the (modified) 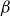 value corresponding to five non-zero wave steepness values 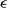 . These calculations use a representative wind speed of 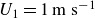 and a relatively smooth surface roughness of 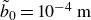 , consistent with the initial generation of waves on a quiescent sea. As done by Miles (1957), the near-surface label 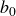 is related to the aerodynamic roughness 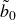 via 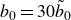 .

Figure 5 shows 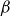 according to (3.19) for a range of wave steepness values. Note that, to reproduce figure 1 of Miles (1957), the 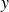 -axis is not on a logarithmic scale (unlike figures 3 and 4) and recall that 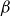 is proportional, rather than equal, to 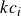 . Nonetheless, 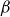 is clearly suppressed as 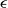 increases, confirming that the growth rate reduction with steepness also holds for the logarithmic profile. Other features also persist: increasing 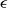 lowers the peak value of 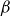 , shifts the wavenumber corresponding to that maximum and narrows the instability window such that it terminates at progressively smaller 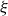 . We describe these shifts in terms of 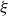 rather than 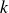 because 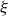 is not necessarily monotone in 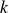 ; it depends on 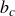 , which itself is a function of 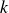 through 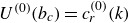 . Although the general suppressive trend is the same for both background profiles, the relative deformation of the curves (peak shifts and cutoff shifts) differs, as expected due to the different near-surface structure of the profiles. In the logarithmic case, shear and curvature are concentrated near the cutoff at 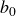 (scaling with 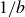 and 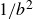 , respectively), so the critical-layer contribution is sensitive to how close 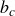 is to 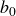 . Since the corrections due to the wave-induced mean flow are intensified near the surface, waves whose corresponding critical layer lies closest to 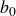 are preferentially suppressed. In contrast, the double exponential profile has bounded near-surface shear and curvature, which decay exponentially away from the interface on the scale 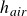 ; consequently, this decay scale controls the steepness-dependent shifts.

### 3.4. Comparison to experimental data

Importantly, (3.19) enables a direct comparison of the theoretical correction to empirical data, offering a new explanation for the observed steepness dependence of growth rates. Peirson & Garcia (2008) systematically investigate the significance of wave steepness for wave growth by combining new wave-tank experiments with several reanalysed datasets (1967–1998). Their formula for 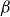 ((7) therein) is given by
(3.21)

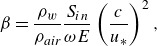

where 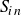 is the energy input from the wind, 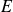 is the local total energy density and 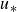 is the friction velocity. While Miles has 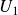 in place of 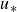 , the logarithmic profile implies 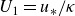 (Miles 1957, (5.3*b*) therein), where 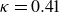 is the von Kármán constant; we therefore rescale 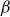 calculated via (3.19) by 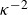 .

**Figure 6.**
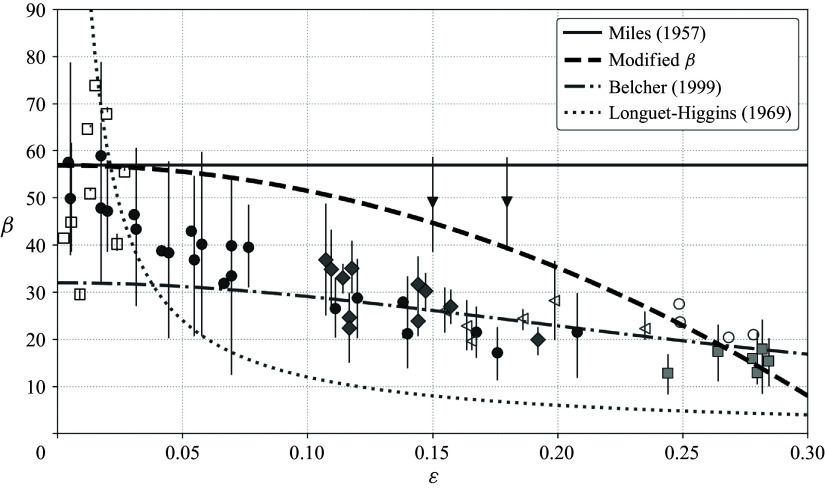
Comparison of the original Miles 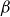 , the modified 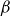 (3.19) and the experimental compilation of Peirson & Garcia (2008) (their figure 6), against wave steepness 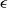 . Symbols and error bars are digitised from Peirson & Garcia (2008), with each marker type denoting a different set of experiments. The Miles baseline (solid curve) and the modified growth parameter (thick dashed curve) are evaluated at fixed background parameters 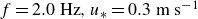 and 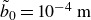 (as in figure 5, 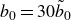 ). Dash-dotted and dotted curves show the reference trends of Belcher (1999) and Longuet-Higgins (1969), respectively, as discussed by Peirson & Garcia (2008).

**Figure 7.**
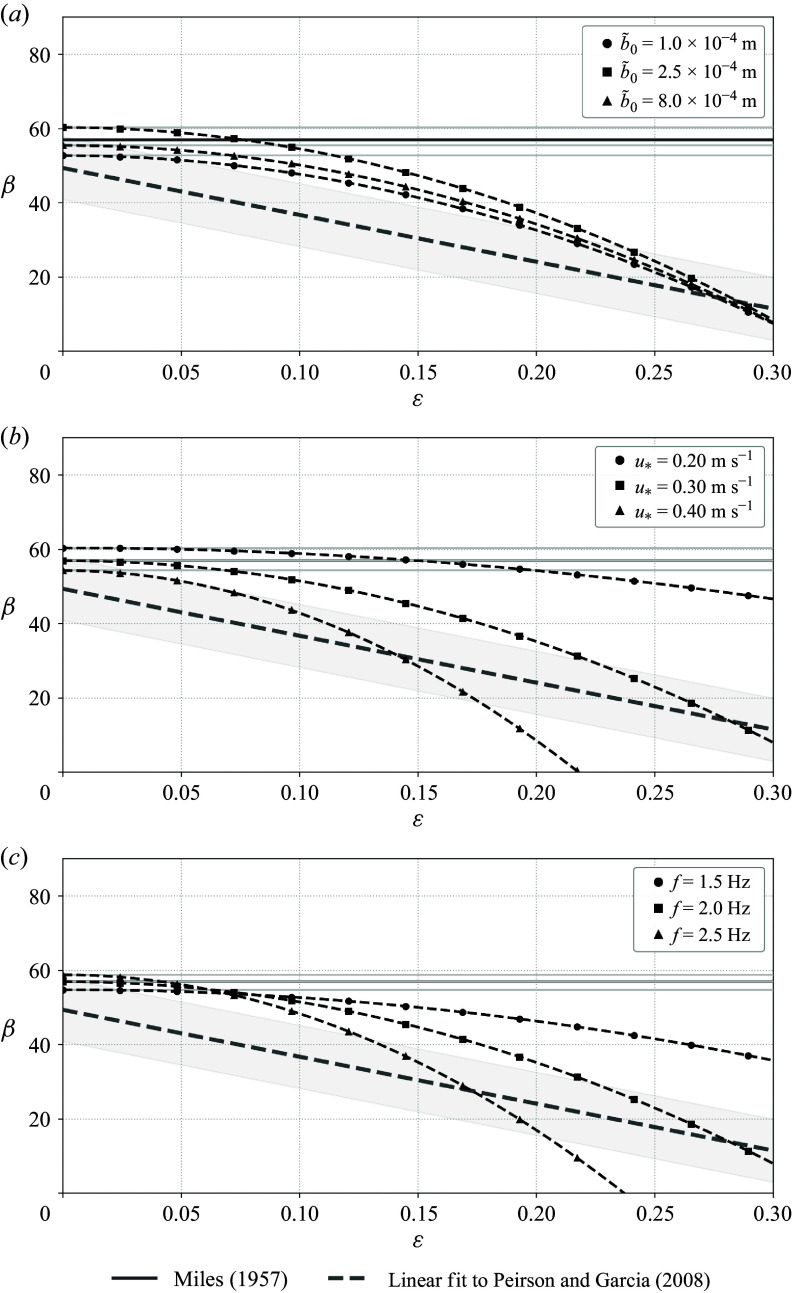
Sensitivity of the 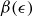 trend calculated according to (3.19) and displayed in figure 6 to the background parameters in the logarithmic profile, with the corresponding Miles 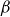 for each set of parameters and a linear fit to the Peirson & Garcia (2008) data plotted for reference. In each panel, the Miles (1957) baseline (solid, light grey) and corresponding modified prediction (black dashed) are compared with the linear fit to the mean trend reported by Peirson & Garcia (2008) (long dashes; shaded bands show one standard deviation of variation calculated from the data, on either side). (*a*) Dependence on aerodynamic roughness parameter 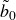 for fixed 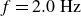 and 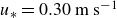 ( 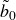 values are as marked in the legend). (*b*) Dependence on friction velocity 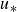 for fixed 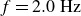 and 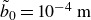 ( 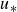 values are as marked in the legend). (*c*) Dependence on wave frequency 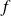 for fixed 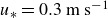 and 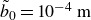 ( 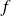 values are as marked in the legend).

Because the experiments analysed by Peirson & Garcia (2008) originate from distinct studies, the background parameters span a broad range: friction velocities 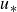 vary from roughly 0.2 m s 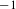 to over 1.0 m s 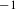 and the intrinsic frequencies 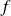 of the mechanically generated waves vary from approximately 0.5 to 6.0 Hz. Consequently, the set of empirical data could be considered as lying on a family of curves rather than a single curve, as (3.19) depends on these different parameters. To assess the general steepness trend rather than predicting individual data points (which can also be subject to large error bars), figure 6 evaluates (3.19) using fixed background parameters within the experimental range: aerodynamic roughness 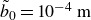 , friction velocity 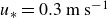 and 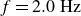 . The sensitivity of the trend to these specific choices is examined in figure 7.

A comparison of the theoretical curve to experimental data demonstrates that the correction due to the wave-induced mean flow (3.19) again yields decreasing wave growth with wave steepness, which captures the qualitative steepness dependence of the data and closely matches the measurements in magnitude over much of the steepness range (figure 6). Although the growth rate is often within the reported error bars at both high and low wave steepness values in figure 6, the fit between theory and experimental data could be more robust if the growth rate were calculated with respect to the background parameters used in each individual experiment, rather than a fixed set. However, not all experiments were reported with specific values of each parameter (as described by Peirson & Garcia (2008) and references therein). Even though the prediction shown in figure 6 is thus not fitted to individual data points, the growth rate modification shows a remarkable correspondence with the observed trend of growth suppression.

Moreover, a calculation of the (approximate) modified growth rate across a range of realistic parameter values (figure 7) demonstrates that in all cases, the modification yields a strict suppression relative to the original Miles growth rate evaluated at the same background parameters. While the magnitude of the suppression depends on parameters – and can even result in no growth at very high steepness given certain parameter values (e.g. high 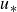 or high 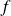 ) – the reduction in growth with increasing wave steepness is seemingly universal. That the modified prediction can reproduce not only the trend, but also the magnitude of experimental results reasonably well may be somewhat surprising given that the present calculation does not include any effects of viscous or turbulent losses, unlike the data from Peirson & Garcia (2008). We also note that the sensitivity to background parameters shown in figure 7 is consistent with the mixed performance of the original Miles-type predictions across experiments (e.g. van Gastel *et al.* 1985 or Wilson *et al.* 1973). Since the modified growth rate equals the original Miles growth rate at 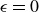 , its correspondence with experimental data is naturally limited by that of the unmodified growth rate. The Miles (1957) prediction itself of course has no explicit dependence on wave steepness and therefore does not reflect the decreasing trend, though it can agree with the magnitude at low steepness.

Despite the obvious dependence on background parameters (figure 7), it is possible to formulate a very simple estimate of the relative change in the growth rate, 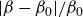 , to assess the practical significance of the wave-slope dependent correction in experimental or observational contexts. This deviation is given by the 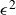 term in (3.19); simplifying with 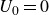 and 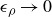 , its magnitude approaches 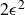 . Wave steepness values in developing seas typically range from 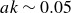 – 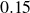 (e.g. Donelan, Hamilton & Hui 1985), but can reach 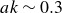 – 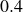 in strong forcing or near-breaking regimes (Perlin, Choi & Tian 2013). Applying this rough estimate, a wave steepness of 0.15 yields a 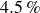 effect and a wave steepness of 0.3 yields an 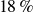 effect, which is further amplified by the exponential nature of wave growth.

Peirson & Garcia (2008) interpret the 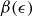 trend by appealing to different mechanisms in different steepness ranges, as marked in figure 6. For the higher-steepness regime, they compare against Belcher’s framework, though this requires tuning the tangential-stress contribution to match observations. While this representation consequently provides a reasonable match at high steepness, it is a poor representation at low steepness, so they instead attribute strong growth in that regime to the Longuet-Higgins ‘maser-like’ mechanism. In contrast, figures 6 and 7 show that the observed trend can alternatively be accounted for across the steepness range with a single Miles-type mechanism. Although this monotonic suppression is clearly evident across the many experiments compiled by Peirson & Garcia (2008), the underlying trend could be obscured by other factors in other empirical datasets. In studies such as that of Peirson & Garcia (2008), ‘growth’ is inferred from net wave-energy evolution. However, Grare *et al.* (2013*b*) find wind-input levels that exceed the net growth rates assembled by Peirson & Garcia (2008), implying that substantial turbulent effects cause this residual metric to underestimate true wind input, especially when breaking is frequent. Extrapolation biases in drag estimates, linked to viscous-stress decreases near the interface (Buckley *et al.* 2020), could also make this relationship less apparent. Irrespective of the various observational conditions that may dilute the steepness dependence, a specific physical mechanism driving the suppression can be identified, as shown through the integral momentum budget formulated in the next section.

### 3.5. Momentum budget

Having demonstrated that the growth rate modification due to the wave-induced mean flow consistently acts to suppress the instability across different background profiles, we now seek to physically interpret this effect by formulating a momentum budget. Expressed in the unapproximated Lagrangian coordinates (2.4), this budget emphasises that the critical-layer interaction is not governed by the Eulerian background shear alone, but by the coupling between the total phase speed 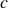 and the total Lagrangian mean flow 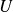 :
(3.22)

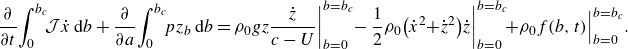

In (3.22), 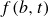 is a constant of integration determined by the phase-averaged pressure at the surface. This budget results from integrating the momentum equation and using an alternative form of pressure which can be derived from the momentum equations (see Appendix E),
(3.23)

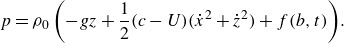

The first term on the left-hand side of (3.22) represents the change in momentum density over time, while the second term on the left-hand side is momentum flux. Physically, the second left-hand term accounts for redistribution of momentum laterally within the layer between the surface and the critical level. The right-hand side accounts for the net momentum input across the boundaries at the surface ( 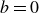 ) and the critical level computed in § 2 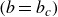 , which is the physical manifestation of form drag. This form drag is split into two components, originating from the hydrostatic and non-hydrostatic components of the pressure (3.23), respectively. The strength of the forcing associated with the first component is proportional to the wave amplitude via 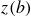 ; however, this is modulated by the key phase shift between wave motion and the pressure field. The second component of the form drag takes the form of a vertical flux of kinetic energy, representing the work done by that pressure component at the boundaries.

Crucially, (3.22) reveals that the critical-layer interaction is governed by the coupling between the total phase speed 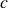 and the total Lagrangian mean flow 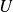 . The suppression of wave growth with increasing wave slope, reflected in both the modified growth rate (figures 3, 4 and 5) and the experimental comparisons (figures 6 and 7), can likewise be seen as a direct consequence of the wave’s nonlinear feedback on its own growth via (3.22). The dependence of the growth rate on wave steepness emerges from the asymptotic expansion; the well-known 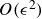 scaling of the wave-induced mean flow results in a growth rate modification of the same order, providing a causal link between steepness and the suppression. While at linear order the critical layer (defined as 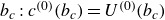 , as it is not necessarily the case that there is any 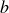 such that 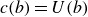 ) resonance is perfect and the momentum transfer is maximally efficient, the higher-order induced mean flow creates a mismatch between the total phase speed and Lagrangian mean flow ( 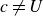 in general). This detunes the resonance and ultimately results in less efficient momentum transfer. Note that this mean-flow mechanism is distinct from a simple alteration of the surface current (as considered in prior studies); it modifies the Lagrangian flow profile without changing the overall shear of the background Eulerian flow. Thus, while the observed growth suppression (Peirson & Garcia 2008) has been explained as a shift from a highly efficient, extrinsic ‘maser-like’ mechanism (Longuet-Higgins 1969) dominant at low steepness, our analysis shows it can be understood as an inherent consequence of the wave’s own dynamics.

## 4. Discussion and conclusions

Our Lagrangian analysis of the Miles instability reveals the key role Lagrangian drift, including the wave-induced mean flow, plays in modifying the growth of wind-generated waves. In particular, the Lagrangian perspective makes evident that the wave-induced current modifies the resonance mechanism upon which the fundamental instability relies. Our analyses, assuming idealised but realistic background wind profiles, demonstrate how the growth rate modification, which depends on the wave-induced mean flow, leads to a suppression of growth with increasing wave steepness. This finding points to an intrinsic self-regulatory mechanism within the Miles critical-layer resonance framework governing the nonlinear evolution of the instability.

Our finding that wave growth can be suppressed by its own induced mean flow is compatible with and offers a new perspective on prior observations of wave growth from laboratory and field studies. As demonstrated by the momentum budget in § 3.5, this feedback acts as an intrinsic self-regulatory mechanism: the wave-induced mean flow detunes the critical-layer resonance, reducing the efficiency of momentum transfer as steepness increases. This result offers a unified theoretical explanation for the steepness-dependent growth rates observed in laboratory and field studies, obviating the need to invoke distinct mechanisms across different steepness regimes.

By remaining entirely within the Lagrangian frame, our formulation of the momentum budget (3.22) most faithfully aligns with Lighthill’s (1962) parcel-based argument, wherein displaced fluid parcels create an asymmetric pressure field that exerts a net force on the wave. The Lagrangian perspective highlights the viewpoint that the instability results from a coupling between different wavy fluid layers: the surface wave itself ( 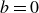 ) and the wave-like motion of fluid parcels within the shear flow (at each level in 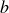 ). At the critical layer, leading-order resonance makes this coupling particularly effective, generating the phase shift between the interior pressure field and the surface elevation. This detailed agreement between the mathematical formalism and the physical intuition underscores the power of the Lagrangian approach for analysing shear instabilities. Indeed, even the particle trajectories themselves (figure 1) provide a direct visual for the instability mechanism: purely circular orbits, corresponding to zero Lagrangian mean flow, would never develop the phase shift necessary for instability.

More broadly, this work establishes a framework for analysing shear instabilities entirely in the Lagrangian frame. Unlike traditional Eulerian analyses that can obscure the particle dynamics central to physical arguments such as those of Lighthill (1962), the Lagrangian approach makes them explicit. This methodology is well suited for revealing the impact of wave-induced flow and can be readily applied to other canonical shear flow problems to gain new physical insights.

While the present analysis focuses on the self-induced flow of the growing mode, the Lagrangian formulation makes clear that the total Lagrangian drift governs the instability, regardless of its origin. Although derived in an idealised, deep-water, neutrally stratified, monochromatic setting, our asymptotic analysis to 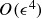 demonstrates that the growth-rate modification depends on the total second-order Lagrangian mean-flow profile 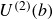 . In this study, 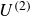 was taken to be the wave-induced mean flow, but the derivation of (3.3) remains valid for any specified mean flow satisfying the generic assumptions of the Miles set-up (as described at the end of § 2.1). Thus, the framework naturally extends to mixed seas by defining 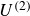 to consist of both wave-induced and background contributions. The net impact on wave growth would then depend on the sign, magnitude and vertical structure of this total higher-order mean flow. For example, a following swell adds positive drift, which would enhance the suppression of wind-sea growth; conversely, an opposing swell subtracts drift, which would reduce suppression. In a directional wind sea, directional spreading reduces the net downwind projection of wave-induced quantities; for equilibrium-range components, Phillips (1985) argues that the directional distribution is broad, implying a reduced net downwind component relative to a purely unidirectional idealisation. However, because the net Lagrangian flow depends on the specific geometry of the sea state – and can be locally enhanced by wave focusing (Blaser, Lenain & Pizzo 2025) – the magnitude of this feedback in the open ocean is difficult to predict *a priori*. Nevertheless, the fundamental result remains: the instability is sensitive to the total Lagrangian mean flow.

The conclusion that wave growth is governed by this total Lagrangian drift connects directly to observational work, as this is precisely the quantity measured by remote sensing techniques that invert for upper ocean currents based on Doppler shifting of surface waves (Pizzo *et al.* 2023). Consequently, observational data gathered via these methods already capture the wave-induced feedback shown here to modify growth. This provides a direct route to reconciling long-standing discrepancies between theory and field measurements by incorporating observational data that measure the full Lagrangian flow. The physical insights enabled by the nonlinear stability analysis thus offer a pathway for refining wind stress parametrisations, particularly for steeper waves or in high-shear conditions where the higher-order modification is most significant.

Additionally, although not addressed in the two-dimensional framework of this paper and its predecessors, the effect of spectral broadening and wave–wave interactions can be considered within this framework, as they affect the local mean drift and do not generally eliminate coherent wave–air coupling altogether. Observations of wave–coherent airflow in both laboratory (Grare *et al.* 2013*b*, 2018) and open-ocean (Hristov, Miller & Friehe 2003) settings confirm that these patterns persist in broadband seas, retaining the characteristic amplitude and phase transitions across the critical layer. Accordingly, random relative phases between different spectral components need not eliminate phase-coherent wave–air coupling for a given component; instead, they primarily modulate which components carry the strongest instantaneous forcing. Nonlinear wave–wave interactions mainly enter by redistributing spectral energy across frequencies and directions; classical weak-interaction theory treats this transfer as a leading-order term that can be comparable to wind input and dissipation in spectral balances (Hasselmann 1962; Phillips 1985). In our framework, such redistribution could strengthen (or weaken) suppression where it increases (or decreases) local drift, without removing the underlying coupling mechanism.

Moreover, this framework may be readily extended to incorporate other environmental factors, which could further refine the calculation of wave growth rates and thus even better alignment with observational data. Although we focus on deep-water conditions, finite depth is known to modify Miles-type growth and can produce a depth-limited regime in which linear growth rates approach zero as waves become sufficiently long relative to the depth (Montalvo *et al.* 2013); extending the present Lagrangian-mean-flow feedback to include a bottom boundary is left for future work. Atmospheric stability likewise alters the background shear profile and can thus shift the critical-layer height (Hristov & Ruiz-Plancarte 2014); in our formulation, accounting for this is naturally handled through the resulting change in the mean-flow profile entering the solution for the modification, (3.3).

Our findings emphasise a key point: the wind interacts with the Lagrangian, rather than Eulerian, mean surface flow. This has implications for the concept of ‘relative wind’, which is central to air–sea interaction. For instance, the ‘eddy killing’ mechanism, in which wind extracts kinetic energy from mesoscale ocean eddies, is a significant dissipation pathway in the ocean’s energy budget (Rai *et al.* 2021). The observed spatial variability of the wave field (e.g. figure 2 of Lenain *et al.* 2023) implies a corresponding variability in this feedback due to the wave-induced component of the Lagrangian drift – the quantification of which remains a topic for future work. A broader implication of this work is therefore that the wave-induced component of the surface flow may be necessary to include when accurately modelling the atmospheric response to ocean dynamics.

## Appendix A. Mathematical details of the linear stability analysis

When expanding  and  asymptotically as in (2.7a)–(2.7c), we can also expand the Jacobian (2.1) and the vorticity (2.3) in the same fashion, so that

(A1a)

(A1b)

To derive the Rayleigh equation, we first find the linear governing equations by substituting (2.7*a*)–(2.7*c*) into (2.2*a*)–(2.2*b*). Note that this portion of the derivation of the instability growth rate (deriving the Rayleigh equation) is not novel and was done by Bennett (2006). The linearised equations are given by

(A2a)

(A2b)

(A2c)

where

(A3)

Note that (A2*c*) is mass continuity;  for all time and  . Differentiating further,

(A4)

Since  , we conclude

(A5)

We now apply the change of variables (2.8*b*). After using (A5) and the chain rule, i.e.  (where this derivative refers to differentiating with respect to the second slot), etc., we obtain exactly the Rayleigh equation (but in Lagrangian coordinates), (2.9).

The Rayleigh equation is used to derive the identity for  , which is used when computing the asymptotic approximation (2.22). The identity (2.23) is analogous to that in the appendix of Young & Wolfe (2014), but is included here for completeness. By multiplying the Rayleigh (2.9) by  , integrating from  to  , and using integration by parts, one finds that

(A6a)

(A6b)

Only the integrand of (A6*a*) has a singularity, which is at  , because we assume  is in the air (  ). To avoid the unphysical singularity, we consider a near singularity and introduce the complex phase speed (2.20). We then approach the singularity by taking  . We compute that

(A7)

Then we can use the following identity (which can be seen from the relationship of the pdf of a Cauchy distribution and the delta function, though Miles (1957) does this using residue calculus):

(A8)

The  in the numerator of (A8) is because of that extra factor in the numerator, while the denominator  arises from the change of variables formula for the Dirac delta.

## Appendix B. Mathematical details of the nonlinear stability analysis

The dispersion relation (3.1) that describes the role of the wave-induced mean flow on the instability growth rate results from considering the system at third order in the wave slope. We first find the dispersion relation at both linear order (2.15) and order  . We will consider the case originally considered by Miles (1957) and neglect the effects of surface tension, setting  . The task then reduces to tabulating the pressure jump at each order, which requires computing each term in (2.6). Obtaining informative results requires significant simplification of this expression, which requires enforcing the conservation of mass and vorticity at each order.

Since we must expand  , we rewrite the expansions of  (2.7*a*) and  (2.7*b*) in terms of wave slope  in more detail. Through second order,

(B1)

We will not *a priori* expand  or  and we will neglect the possible dependence of  on  for now, though this will later be accounted for when we explicitly apply a generalisation of the change of variables (2.8*a*)–(2.8*b*). Otherwise expanding,

(B2a)

(B2b)

Here,  for ease of notation. In addition to one more  contribution from the second-order terms (i.e.  due to  , the higher order oscillatory terms will not provide any additional contributions through third order. We do not expand further because at second order, we will see  is required for conservation of vorticity.

Starting at order  , the conservation of mass condition,  , simplifies to

(B3)

where the use of primes throughout this section will always denote differentiation with respect to  . The condition (B3) implies  . It can also be computed that  .

First-order conservation of vorticity requires

(B4)

which implies  . Using these facts, we can evaluate each term in (2.6) to obtain the pressure-jump portion of dispersion relation (2.14), after changing variables. The linear pressure is evaluated as

(B5)

Conservation of mass at second order requires that

(B6)

which implies the Jacobian is given by

(B7)

Conservation of vorticity at second order requires

(B8)

and importantly that

(B9)

Condition (B9) is necessary to avoid time dependence of the vorticity, but also eliminates secular growth. Then, the vorticity term (i.e. vorticity multiplied by the Jacobian) is simply

(B10)

Assembling the contributions to the pressure, we find that

(B11)

However, (B11) cannot be used to yield a dispersion relation reflecting the impact of the wave-induced mean flow, so we continue to third order.

Conservation of mass at third order requires

(B12a)

(B12b)

and the Jacobian at this order is  .

Conservation of vorticity at third order requires that

(B13a)

(B13b)

To this point though, we have not considered that we want to make a change of variables such that

(B14)

We would make an analogous change of variables for  etc. as well, but the impact of  on these terms (and the impact of  on  ,  ) only comes in at  , so we do not explicitly consider those contributions here. This is also why we omit  from the denominator of (B14). Note that we neglect any possible dependence of  on  when finding additional terms, as we do not assume a specific form of  *a priori*. If the modified growth rate we calculate is applied to wind profiles that yield  that do depend on  , this amounts to assuming the dominant impact of  in the amplitude is through its appearance in the denominator from the change of variables. Given a particular definition of  , one could also account for the impacts of this term *a posteriori* in (B31), as the term will not be secular. When we expand using (B14), we obtain

(B15)

Solving for  , the additional term in the equation for  (2.7*b*) is then given by

(B16)

In the equation for  , we obtain another term as well,

(B17)

where (B3) implies

(B18)

The extra terms (B16) and (B18) add zero to the Jacobian, leaving (B12*a*) and (B12*b*) unchanged.

At third order, the kinetic energy term in (2.6) is complicated, so we write out the evaluation in more detail here. The kinetic energy term, first without the additional terms due to the dependence of  on  (i.e. (B14)), is

(B19)

Before assembling the contributions to the dispersion relation at this order, recall from first order

(B20)

Since we neglect the effects of surface tension, we intend to set the pressure jump equal to zero. We then see that the secular terms in kinetic energy expression (B19), together with the secular term that would come from  , i.e. as shown in (B2*b*), will contribute zero to the third-order dispersion relation. This is because (B20) in the case of no surface tension implies

(B21)

which would still hold when multiplied by, e.g.  .

When considering the terms arising from the dependence of  on  , and computing the kinetic energy and the gravitational term, the contribution of  and the first term in  will reduce to zero due to the linear dispersion relation. However, the last term in  (B18) remains, and it can be seen that its contribution via the kinetic energy is

(B22)

The remaining contributions to the third-order dispersion relation are (omitting those whose contributions will cancel out, as discussed previously)

(B23)

(B24)

(B25)

(B26)

The pressure is then

(B27)

We can rewrite (B27) using the trigonometric identities

(B28)

(B29)

as

(B30)

Since  and  (and a constant) are linearly independent functions, we can satisfy the pressure-jump condition by equating the coefficient of each term to zero independently. Doing so for the  term reveals that  satisfies the dispersion relation

(B31)

as in § 3.1.

## Appendix C. Inclusion of surface tension effects

Surface tension effects appear on the right-hand side of the dynamic boundary condition, e.g. in (2.10). The curvature effects are given by  , where  is the coefficient of surface tension and  is the curvature at a given point on the water surface. In Lagrangian coordinates, for a fixed time  , the surface is described by the parametric curve  . The curvature for a parametric vector is given by (using the convention of signed curvature)

(C1)

We compute

(C2a)

(C2b)

Upon substituting (C2*a*) and (C2*b*) into (C1), we obtain

(C3)

Note that (C3) is also obtained upon transforming the Eulerian definition of curvature, with respect to the free surface  ,

(C4)

into Lagrangian coordinates directly. We now evaluate (C3) at each order of  , using (B2*a*), (B2*b*), including the additional terms due to the change of variables,  (B18) and  (B16). We obtain, again denoting  :

(C5)

(C6)

(C7)

Notice that the secular term and the term with  are exactly those needed to generalise (B21) so that the contributions from the secular term and  in the dispersion relation still reduce to zero. Then, again due to the linear independence of  and  , the third-order surface tension effects only contribute an extra term of

(C8)

to the right-hand side of (B31), which consequently remains linear in  . Note that the matter of continuity of  at zero is irrelevant to the case  , but may require more careful consideration for more complex background shear profiles. Additionally, the inclusion of surface tension effects yields the solution  per (2.18).

## Appendix D. Explicit formula for the wave-induced mean flow

We derive the form of the (second-order) wave-induced mean flow  – or otherwise stated, the Lagrangian frame analogue of Stokes drift – without making any assumptions on vorticity, by adapting Stokes’ (1847) original argument to Lagrangian coordinates and to the situation in which there is a leading-order Lagrangian mean flow.

Let  denote the deviation of a particle’s  -position from the background state  and  denote the deviation of a particle’s  -position from the background state  . We will find the values of  and  occurring at second order in the wave slope by using the forms of  and  . We compute to first order (omitting order  terms since they already appear in the series expansions for  (2.7*a*) and  (2.7*b*))

(D1)

Inside of the cosine, we approximate  since we are only concerned with order  and lower terms. In this section, we again denote  for compactness. Then, (D1) becomes

(D2)

To first approximation,  and  . Then,

(D3)

Using the double angle identity,

(D4)

Using mass continuity (B3), (D4) becomes

(D5)

Integrating in time, we obtain that the first-order portion together with the new second-order portion is

(D6)

Now repeating the same procedure for  , we have

(D7)

Since  (B3), the order  term in (D7) reduces to zero. Then, upon integrating in time, there is no additional contribution at second order to  , and simply

(D8)

Thus, we can identify  as  . Note that one could have alternatively completed this derivation in Eulerian coordinates, as in the original derivation, and used the relationships (2.24*a*) and (2.24*b*) to obtain the final result. This would be possible because one would only account for the leading order portions (which includes the mean flow in  ) and not the higher order oscillatory terms when doing so, since the additional impact of those (e.g. oscillatory) terms would ultimately only yield  terms.

## Appendix E. Derivation of alternative form of the pressure

To derive (3.23), we start with the full Lagrangian momentum equations, (2.2*a*) and (2.2*b*). Multiplying (2.2*a*) by  and (2.2*b*) by  , and adding, we obtain

(E1)

Dividing both sides by  ,

(E2)

Now, we use the identities  and  (derived from (2.4)) to obtain

(E3)

Thus,

(E4)

Integrating in  , we obtain

(E5)

where  is a constant of integration that can be determined by the value of the pressure at the surface and setting  , where  is evaluated according to (E5).

The momentum budget (3.22) is then derived using integration by parts twice, various identities relating derivatives in  and  derived from (2.4), and (E5).

## Appendix F. Details of the approximation for the case of a background logarithmic profile

To derive (3.19), we must first establish the correspondence between Lagrangian coordinates and the ‘streamline coordinates’ used in the original analysis by Miles (1957). Miles (1957) regarded the vertical variable as a streamline coordinate (i.e. a label for the base-state streamlines, rather than the instantaneous geometric height) and the kinematic boundary condition is applied on a near-surface streamline. In that description, the vertical coordinate plays an analogous role as  does in the Lagrangian frame. It is therefore natural to choose the two label systems to coincide at the level of the base flow, i.e.  and to identify a specific  -value  with Miles’  . We emphasise that  is a particle label and therefore need not coincide with an instantaneous streamline coordinate in the disturbed flow; however, Miles’  is also used only as a base-state streamline label. Accordingly, in this section, we keep the levels of Miles’ approximation fixed and incorporate finite-amplitude effects only as they relate to the modified eigenvalue.

With the coordinates established, we now formulate the general form of  that we aim to evaluate with respect to (3.12). To obtain a formula for  , Miles (1957) first assumes the pressure in the air is proportional to the local wave displacement, and writes it in the form

(F1)

where the surface wave has the form  . It is also assumed that the density ratio  and that the inertial effect of the air is negligible, meaning the real part of the phase speed is not significantly altered. This allows the zeroth-order approximation  . The phase speed  may then be approximated as (3.14) (Miles 1957, (2.9)) and, correspondingly, the normalised growth rate  is approximated as (3.15). Thus,  is obtained via a mixed-order approximation; based on the physical assumptions, the real part of the phase speed is only ever taken as its leading-order (in the small density parameter  ) component, while the imaginary part is taken through linear order. To remain consistent with this, when we now introduce a second asymptotic expansion but in wave slope  to obtain a modified version of (3.15), we likewise consider the imaginary part of the phase speed through higher orders while fixing the real part at (3.16). We thus substitute in  for  in (3.15), so we must evaluate  (3.3) with respect to the background profile (3.12).

Recall that  is such that  . Using (3.12),

(F2)

For convenience, we define the scaled labels

(F3)

Now, analogous to (3.5*b*) of Miles (1957), we define

(F4)

One may then write a non-dimensional Rayleigh equation directly analogous to Miles (1957) and follow the exact same steps to obtain (3.20).

To evaluate the air-side terms in  , first define

(F5)

We adopt Miles’ trial outer eigenfunction and normalise it so that  (equivalently,  :

(F6)

Differentiating (F6) with respect to  and evaluating at  ,

(F7)

Using the chain rule, we can evaluate

(F8)

Substituting in (F5),

(F9)

We may then evaluate

(F10)

Now, we evaluate  and its shear per (3.5), at the near-surface label,

(F11)

(F12)

Within this log-law closure, the second-order amplitude terms  do not contribute at  for the above-mentioned exponential form of  , per (3.4). We assemble, per (3.2*a*) and (3.2*b*),

(F13a)

(F13b)

Evaluating on the water side is simple as  for  . The Rayleigh equation (2.9) reduces to  ; imposing decay as  yields  . Then, from (2.8*b*),  , and hence,

(F14)

Note that in general, the solution would be  ; we have selected  in (F14) since we normalise such that  , invoke the Miles transition-layer assumption  , and assume  is continuous across the interface. On the water side,

(F15)

and one may compute per (3.5) that

(F16)

We may then evaluate

(F17a)

(F17b)

Thus, again using (3.2*a*) and (3.2*b*), we assemble

(F18a)

(F18b)

Substituting (F13*a*), (F13*b*), (F18*a*) and (F18*b*) into (3.3) yields

(F19)

Now, we must find the imaginary part of this calculated  . After expanding  in terms of  , in a similar fashion to (2.17), we obtain a simple form for  ,

(F20)

Substituting (F20) into the Miles mapping (3.15) yields

(F21)

where  is the original (leading-order) normalised growth rate. To obtain the last line of (F21), we used that

(F22)

also per (3.19).

To evaluate (F21), we must then translate  to Lagrangian coordinates, but this is made convenient by the fact that the derivation of Miles (1957) is already in a ‘streamline coordinate’, rather than a purely Eulerian coordinate, following the shape of the interface. Hence, following the exact same mathematical steps as Miles (1957) yields (3.20). All that is left is to explicitly compute and evaluate the derivative, which becomes

(F23)

Thus, per (F21),

(F24)

However, (F24) is written in terms of what is formally the small parameter  appearing in the Lagrangian expansion; i.e. in the present normalisation,  is not *a priori* the geometric steepness  used in experimental growth curves. This is a consequence of fixing the otherwise arbitrary scaling of the Rayleigh eigenfunction by imposing  . Indeed, in the Lagrangian ansatz, the first-harmonic vertical displacement at label  has the form  , so the physical amplitude  of the displacement at the label  is  For the log-law closure with  , we obtain  . Since  is related to  via (F10), the normalisation  also fixes  and the remaining freedom to match the physical amplitude is carried by  . Therefore,  , as we have used it thus far in this section, is related to the physical steepness via  . Of course, the small imaginary portion is irrelevant to  and we replace  by  , giving  . Substituting this into (F24) yields the final form, (3.19), in which  can actually be interpreted as  .
